# Noninvasive Vascular Images for Face Transplant Surgical Planning

**Published:** 2011-12-14

**Authors:** Shigeyoshi Soga, Nicole Wake, Ericka M. Bueno, Michael L. Steigner, Dimitrios Mitsouras, Kurt Schultz, J. Rodrigo Diaz-Siso, Geoffroy C. Sisk, Richard Prior, Sara L. Powers, Jason Signorelli, Camille K. Jania, Bohdan Pomahac, Frank J. Rybicki

**Affiliations:** ^a^Applied Imaging Science Laboratory, Department of Radiology; ^b^Department of Surgery, Division of Plastic Surgery, Brigham and Women's Hospital, Francis St, Boston, MA

## Abstract

**Objective:** Face transplantation replaces substantial defects with anatomically identical donor tissues; preoperative vascular assessment relies on noninvasive imaging to separate and characterize the external carotid vessels and branches. The objective is to describe and illustrate vascular considerations for face transplantation candidates. **Methods:** Novel noninvasive imaging using computed tomography and magnetic resonance imaging over 3 spatial dimensions plus time was developed and tested in 4 face transplant candidates. Precontrast images assessed bones and underlying metal. Contrast media was used to delineate and separate arteries from veins. For computed tomography, acquisition over multiple time points enabled the computation of tissue perfusion metrics. Time-resolved magnetic resonance angiography was performed to separate arterial and venous phases. **Results:** The range of circulation times for the external carotid system was 6 to 14 seconds from arterial blush to loss of venous enhancement. Precontrast imaging provided a roadmap of bones and metal. Among the 4 patients, 3 had surgical clips, metal implants, or both within 1 cm of major vessels considered for surgery. Contrast-enhanced wide area detector computed tomographic data acquired in the axial mode separated these structures and provided arterial and venous images for planning the surgical anastomoses. Magnetic resonance imaging was able to distinguish between the large vessels from the external carotid systems. **Conclusions:** Vascular imaging maps are challenging in face transplantation because of the rapid circulation times and artifact from the initial injury, prior reconstructive attempts, or both. Nevertheless, face transplant candidates require high spatial and temporal resolution vascular imaging to determine those vessels appropriate for surgical anastomoses.

Facial allograft transplantation, that is, face transplantation, belongs to the field of vascularized composite allotransplantation, a relatively new surgical discipline to transplant nonparenchymatous tissue composites. Treatment with conventional procedures such as skin grafting, local flaps, pedicled flaps, and more recently free flaps is often multistaged and ultimately leads to less than acceptable results in the most severely disfigured patients. Face transplantation, in contrast, is a complex but reasonable means of providing ideal restoration to a select group of patients by “replacing” rather than “reconstructing” damaged structures. The procedure replaces missing facial structures with anatomically identical tissues, providing desired functional, aesthetic, and psychosocial benefits far superior to conventional methods.^1^^,^^2^

A recent review^3^ describes the advantages of face transplantation over conventional facial reconstruction and includes a comprehensive assessment of the nonimaging screening and the risks, benefits, and follow-ups after the procedure. In brief, at our institution, face transplantation is considered for potential candidates with defects involving 25% or more of the facial surface area, and/or the loss of one or more main facial units, for example the nose or lip(s). The most common injuries are from gunshot, explosive device, and burn injuries. Candidates with malignancy, infection, and congenital defects are also considered.

Once transplanted, and unless the allograft is removed, patients must submit to a closely monitored lifelong regimen of immune suppression. This regime is associated with severe short- and long-term potential adverse effects, including infection, malignancy, metabolic complications, and organ toxicity. Even with complete compliance to immunosuppression, there remains a risk of rejection and ultimately failure. Fortunately, the world experience with facial transplantation continues to demonstrate promise and unparalleled functional, aesthetic, and psychosocial outcomes.

Determining the best potential candidates is complex, because it is essential to identify individuals who can provide informed consent and for whom the risks do not outweigh the benefits. Thus, selection of candidates involves exhaustive psychosocial and systemic evaluation by a highly experienced, multidisciplinary team of people who can maximize the benefits with respect to the risks of the procedure. This study describes and evaluates one important component of the screening process, namely the preoperative vascular imaging from computed tomographic (CT) and magnetic resonance (MR) acquisitions.^4^^-^8 We also emphasize the critical role of advanced image postprocessing strategies used to delineate the course, caliber, and contrast enhancement patterns of the arteries and veins that will be used at face transplantation.

## METHODS

All subjects signed written informed consent approved by our Institutional Human Research Committee, voluntarily enrolled in clinical trial NCT01281267, and are documented in the US Army Medical Research and Materiel Command's Human Research Protection Office.

### Face transplant candidates

**Figure 1:**
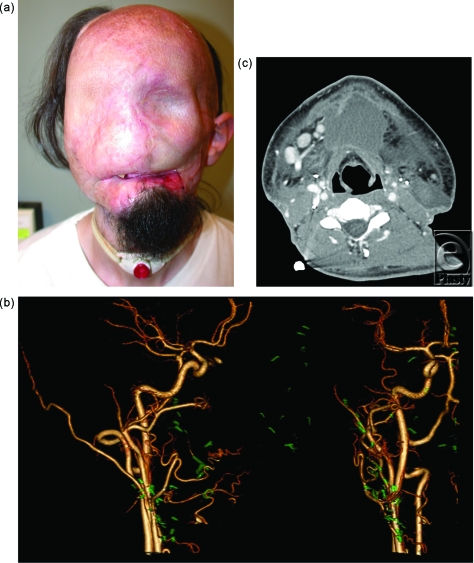
(*a*) Candidate 1 during initial evaluation for face transplantation. (*b*) Pure arterial images shown in 3-dimensional volumes after processing the 320 × 0.5 detector row computed tomographic (CT) images. The metal fragments, predominantly clips from the multiple surgical flaps, are separately segmented and denoted in green to show the proximity between the arteries and metal that must be identified for the surgical dissection. The figure demonstrates right cervical arterial system in 2 different viewing angles. (*c*) Axial slice from CT scan 13 days after face transplantation revealed abnormal fluid collection and subcutaneous edema in the submental region.

Candidate 1 is a 24-year-old man, who had catastrophic loss of facial tissues after high voltage injury. After 20 procedures highlighted by multiple flaps covered with skin grafts, other surgical options were exhausted and the patient presented to our institution (Fig 1a).

**Figure 2:**
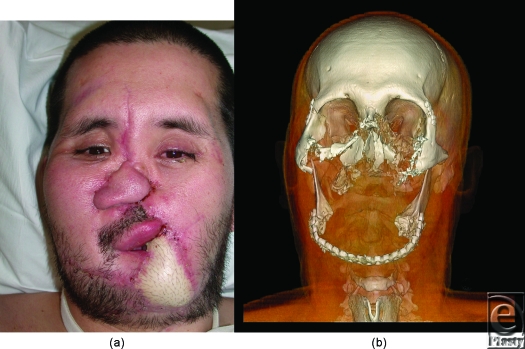
(*a*) Candidate 2 during initial evaluation for face transplantation. (*b*) 3-dimensional volume rendering from the computed tomographic acquisition with windowing to show the bony defects that must be considered in surgical planning.

Candidate 2 is a 36 year-old man, who suffered accidental gunshot wound to the central face. Reconstructive surgical options were exhausted before the patient had an aesthetic appearance acceptable to society, and before oral and breathing functions were adequately restored (Fig 2a).

**Figure 3:**
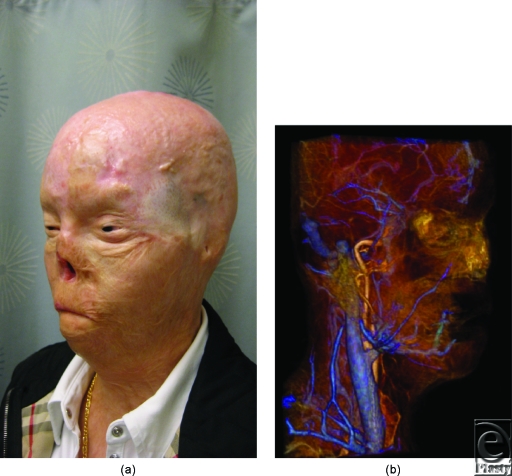
(*a*) Candidate 3 during initial evaluation for face transplantation. (*b*) Venous phase conventional magnetic resonance (MR) venography. In patients without artifact in close proximity to the major vessels considered for anastomoses, both computed tomography and MR imaging demonstrate excellent venous detail.

Candidate 3 is a 30-year-old woman, who had a burn injury to 70% of her body, including the majority of her face (Fig 3a).

Candidate 4 is a 40-year-old man, who suffered a blast injury causing a through-and-through defect of the right cheek and loss of his right lower lip. Multistaged reconstruction ultimately resulted in a satisfactory esthetic outcome but failed to restore oral and sensory functions. Note that photographs are not provided at the candidate's request.

### CT imaging

Acquisition^5^ used a 320-detector row scanner (AquilionOne, Toshiba Medical Systems, Tochigi-ken, Japan) operating in a single-volume axial mode. In other words, helical imaging was not performed; the entire 16-cm craniocaudal field of view was imaged without patient or CT couch movement. Precontrast images were evaluated for volume rendering of bones, and to assess noncalcified structures such as surgical clips, implanted materials, shrapnel, and/or other foreign bodies. Intravenous contrast (iopamidol 370 mg iodine per milliliter, Isovue-370, Bracco Diagnostics, Princeton, New Jersey) enhancement used a power injection (Empower CTA, Acist Medical, New York) system. Because face transplant candidates often have multiple injuries, peripheral intravenous access was challenging. Twenty- or 18-gauge access was obtained, and contrast flow rates of 4 to 6 mL/s was used for dynamic imaging.^9^

Scanner output data were used to estimate the radiation dose from face transplant screening. Radiation from precontrast and contrast-enhanced studies is cumulative. The thyroid gland is within or adjacent to the imaging field of view and must be considered in the CT protocol.^10^^,^^11^

### MR imaging

A 3-Tesla MR (Siemens Trio, Erlagen, Germany) protocol^6^ was based on multiphase or so called “time-resolved”^12^ acquisitions. External carotid and branch arterial and venous imaging used gadolinium contrast (MultiHance, Bracco Diagnostics, Princeton, New Jersey) enhancement. From the multiple phases over time, the best pure-arterial and pure-venous images were extracted for review and image postprocessing. These Magnetic Resonance Angiography (MRA) acquisitions do not deliver ionizing radiation.

### Image postprocessing

All CT and MR images were exported in DICOM format to a dedicated image postprocessing workstation (Vitrea fX, Version 6.1; Vital Images, Minnetonka, Minnesota).

#### Multiplanar reformations

Because image display in standard axial, coronal, and sagittal planes does not adequately depict the complex relationship between structures, multiplanar reformations were used to manipulate off-axis imaging planes at high spatial resolution so that spatial relationships between arteries, veins, bones, and metallic fragments could be defined. Multiplanar reformations maintain the spatial resolution near or at that acquired for both CT and MR. However, this results in a large number of output images, and thus, while these images are essential for interpretation, they were used less for communication between the radiologist (F.R.) and surgeon (B.P.) for preoperative planning.

#### Maximum intensity projections

These views condense larger volumes of information into slabs of variable thickness.^13^^,^^14^ Maximum intensity projections have the advantage of fewer total images. However, the disadvantage is that clinically relevant information can be lost in the maximum projection of data as the slabs become thicker. The fewer images and thus smaller data sets were more amenable to communicate findings, and these projections can be viewed in a cine loop that can be stopped and advanced manually, enabling a visual, subjective assessment of the dynamic blood flow of small vessels over time. This 3-dimensional imaging over multiple time points is used to assess flow to facial structures. This has distinct advantages over catheter-based angiography that yields only planar imaging over time.

#### Volume rendering

This method extracts isosurfaces from volumetric data, based on attenuation for CT and signal intensity for MR. These volumes were essential for optimal communication, as facial surfaces can overlay vascular (or other crucial) structures with any level of intensity. Volume rendered images are routinely used for organ transplantation planning.^15^

### Quantitative analyses

For all 4 patients, the circulation time was recorded from the cine CT data and defined as the time elapsed between the initial enhancement of the external carotid artery and the loss of enhancement of the external jugular vein. The CT timing was used for MR triggering. All nonosseous metal was characterized on the pre-contrast CT images; the peak enhancement images from the subsequent contrast-enhanced images were used to determine the spatial relationship between the vessels considered for surgery and metal in the preoperative facial tissues.

## RESULTS

### CT imaging

Computed tomographic angiography provided outstanding visualization of arteries (Fig 1b) and veins (Fig 3b) for planning complex anastomoses. Eighteen- or 20-gauge peripheral venous access was achieved in the antecubital or wrist station in all patients. The CT suite “room” time (door to door) was less than 45 minutes for all patients. Image acquisition for arterial and venous images was less than 1 minute. All images were acquired with less than 100 mL of contrast material, and the estimated, effective total radiation dose was less than 10 milliSeverts for all patients.

### MR imaging

**Figure 4:**
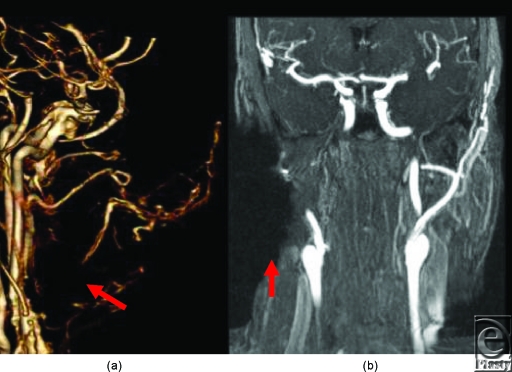
(*a*) Arrow points to a region of signal void artifact from surgical clips. Artifacts significantly degrade conventional MRA image quality and limits the broad application of MRA, as candidates often have metallic implants from prior reconstructions. (*b*) Note large region (*arrow*) of marked signal loss due to implanted metal artifact.

Metallic artifacts tend to be more severe in MR than in CT^6^; however, subtracted time-resolved MRA images were viewed in multiple projections to provide large vessel data similar to CTA for surgical planning. Assessment of distal, diminutive vessels was limited, primarily by the lower spatial resolution of MR, and secondarily by greater magnitude of metal artifacts (Fig 4).

### Image postprocessing

#### Multiplanar reformations

Preserved, superior spatial resolution enabled separation of important anatomic relationships that can be lost with thick maximum intensity projections. The total number of images was far greater for thin-section multiplanar reformatted images, and this had the secondary effect that individual images have lower signal to noise ratio, based on the fact that there is less tissue (ie, facial structures) in thinner slices.

#### Maximum intensity projections

Variable thickness slabs illustrated differing tissue volumes. Higher signal enabled rendering of large slabs of tissue from any projection. For example, the complex course of the facial artery was demonstrated in relationship to the bone and previous reconstructions. When too thick, maximum intensity projections suffered from volume averaging because only the structure with the highest intensity was portrayed.

#### Volume rendering

Soft tissues and bony defects used for allograft design were best depicted with volume rendering (Fig 2b). Photographs and other digital imaging after conventional reconstructions have the limitation that they show only superficial structures such as skin grafts and thus fail to provide information regarding important deep tissue and vascular defects. Volume-rendered planning for bone reconstructions were critical for the procedure. Three-dimensional plus time assessment of vascular enhancement was achieved by cine fusion of individual volume-rendered images during arterial and subsequent venous enhancement.

### Quantitative analyses

The circulation times ranged between 6 and 14 seconds from 3-dimensional imaging frame counting. Regarding the evaluation of nonosseous metal, 3 of the 4 patients had shrapnel, implanted devices, or both, which coursed within 1 cm of a major vessel considered for anastomoses. These were best identified on multiplanar reformatted images. These spatial relationships were considered highly valuable for presurgical planning.

## DISCUSSION

Transplantation candidates have disfigurement of at least 25% of the facial surface area, which includes the loss of function and/or aesthetics. Candidates undergo comprehensive screening for transplant qualification, after which they are placed on the transplant waitlist. This report describes the large role of vascular considerations in screening, and demonstrates the close proximity of high attenuation structures (eg, shrapnel) to key vessels that must undergo dissection during the transplant surgery. We also report rapid transit times for the circulation. Very short times can be from vascular shunting either as a result of the injury or prior interventions.

Noninvasive imaging has largely replaced catheter-based angiography. This is similar to most other transplant surgery planning. In addition to avoiding the risk of complications from introduction of a catheter, CT and MR imaging yield 3-dimensional assessments over time that are not possible with other imaging modalities. A facial allograft (including skin, underlying soft tissues, cartilage, bones, nerves, arteries, and veins) is designed on the basis of subject's defects; superficial structures are readily assessed by direct examination, but deeper defects are largely determined by imaging. When a donor is found, simultaneous to the procurement of the facial allograft, the recipient's vessels and nerves are dissected in preparation for anastomoses. Ischemia time of the allograft is thus minimized by rapid anastomoses and allograft reperfusion. This critical part of the surgical procedure is planned with careful investigation of the presence, course, caliber, and contrast enhancement of the recipient's external carotid and its branches, both arterial and venous. Vascular anastomoses are followed by neurorrhaphy and subsequent bony and soft tissue fixation.

On the basis of our experience to date, the superior spatial resolution of CT, combined with the fewer metallic artifacts, has made CTA the first line modality for screening. Selection of recipient vessels is determined by factors such as diameter match with donor vessels and length of allograft pedicle; CT findings have excellent correlation with surgical findings. Our protocol is based on 320-detector row CT technology that spans a 16-cm (320 × 0.5 mm) craniocaudal field of view. The gantry can be angulated up to 15 degrees, a potentially important adjustment for radiation dose considerations. For example, in a patient with at least partial vision, the field of view can be oriented along the infraorbital rim so that the orbits are not directly included in the main radiographic CT beam. Because cumulative radiation exposure can induce cataracts, this is particularly important because imaging is often performed for follow-up and for potential postoperative complications (Fig 1c).

Another substantial advantage of this CT technology is that the 16-cm coverage has, for all candidates to date, spanned the entire planned facial allograft. Thus, patients can be imaged axially, or with no table motion, as opposed to helical CT where the patient is moved in and out of the CT gantry during scanning. With no patient motion, transplant candidates are imaged over multiple time points during contrast enhancement to ensure that both arterial and venous maps can be generated for surgical planning. These methods thus resemble time-resolved MR angiography.^12^ Recent reports indicate additional diagnostic value of 3-dimensional imaging over time for vessels that are small or have a narrow temporal window such as lower extremity or pulmonary vessels.^16^^-^18 Given the short transit times in our patients, the ability to separate arteries and veins is considered highly valuable for surgical planning.

Comparing the 2 modalities, CT has superior depiction of small vessels but has the disadvantage that it delivers ionizing radiation that, in theory, can induce a fatal malignancy. Moreover, the thyroid gland is near, or in, the imaging field of view for face transplant screening angiography. To minimize radiation, electrocardiographic gating^19^ is not used, and the filed of view is often tailored to less than 16 cm, for example, 12 or 14 cm for candidates with a smaller planned allograft.

Image interpretation and communication between the radiologist and surgeon uses all available methods of image postprocessing and display. For example, a surgical clip near an important contrast-enhanced vessel is likely to be obscured on the MR data by susceptibility artifact. On the CT images, the spatial relationship is best defined by the thin, higher spatial resolution multiplanar reformatted images. These can then be used to create higher signal to noise ratio maximum intensity projections, with a slice thickness tailored to best show the structures while maintaining distinction between structures. These images are then used to orient specific axes for viewing cine runs that subsequently show the full course of the vessel(s) in question. This iterative sequence over all potential metal and vessels is a critical vascular consideration in screening face transplant candidates to minimize or eliminate potential catastrophic complications at surgical dissection.

There are several limitations to our study. First, CT and MR findings in face transplant candidates cannot be meticulously correlated with conventional angiography findings. However, we confidently report in these patients that there were no clinically significant discrepancies between preoperative vascular maps and vessels found at surgery. Even though it has the highest spatial and temporal resolution available, catheter angiography is no longer clinically indicated and CT has become the general reference standard for transplant surgery planning. The second limitation is the small number of subjects. However, the surgery itself, particularly for full face transplants, is very nascent, and thus there are no larger population studies, although we expect on the basis of initial success with several patients that the field will grow.

We speculate that the pretransplant evaluation of living donors using noninvasive imaging techniques such as CTA and MRA will allow the simultaneous evaluation of arteries and will be useful for tailoring anastomoses based on the size and length of available vessels. Future work will generate complex models based on these images that are likely to be useful for surgical planning and training. Noninvasive images acquired over time can also be used to analyze blood flow in vessels, shear stress on the endothelium,^20^ the importance of contrast enhancement differences,^21^ and metrics of preoperative tissue perfusion. These novel imaging strategies can then be used postoperatively, both for screening and for potential tissue rejection characterized by early changes in the vessel wall that can, in theory, be assessed by ultra-high resolution imaging methods.^22^^,^^23^

In summary, CT angiography provides presurgical planning that includes key vascular considerations. In particular, the spatial relationships for facial tissues must be fully characterized, and this analysis is usually complicated by the patients' initial injury as well as early reconstructive procedures. The majority of our patients have nonosseous metal near the vessels considered for anastomoses as well as rapid arterial flow and venous return, and thus the assessment requires meticulous 3-dimensional image acquisitions over time and after processing. Radiation considerations are important, making MR-based methods attractive for future development preoperatively and sequential postsurgical evaluation. In addition, new methods will be developed, such as imaging of potential rejection, as clinical experience expands.

## Acknowledgments

The work was supported by US Department of Defense contract W911QY-09-C-0216.
